# Exploring the dimensions of patient experience for community-based care programmes in a multi-ethnic Asian context

**DOI:** 10.1371/journal.pone.0242610

**Published:** 2020-11-25

**Authors:** Chuan De Foo, Yan Lin Tan, Pami Shrestha, Ke Xin Eh, Ian Yi Han Ang, Milawaty Nurjono, Sue-Anne Toh, Farah Shiraz

**Affiliations:** 1 Saw Swee Hock School of Public Health, National University of Singapore, Singapore, Singapore; 2 Singapore Population Health Improvement Centre (SPHERiC), National University Health System, Singapore, Singapore; 3 Regional Health System Office, National University Health System, Singapore, Singapore; 4 Centre for Health Services and Policy Research (CHSPR), Saw Swee Hock School of Public Health National University of Singapore, Singapore, Singapore; 5 Health Services Research, Changi General Hospital, Singapore Health Services, Singapore, Singapore; 6 Yong Loo Lin School of Medicine, National University of Singapore, Singapore, Singapore; ESIC Medical College & PGIMSR, INDIA

## Abstract

**Introduction:**

The aim of this study is to explore patients’ experiences with community-based care programmes (CCPs) and develop dimensions of patient experience salient to community-based care in Singapore. Most countries like Singapore are transforming its healthcare system from a hospital-centric model to a person-centered community-based care model to better manage the increasing chronic disease burden resulting from an ageing population. It is thus critical to understand the impact of hospital to community transitions from the patients’ perspective. The exploration of patient experience will guide the development of an instrument for the evaluation of CCPs for quality improvement purposes.

**Methods:**

A qualitative exploratory study was conducted where face-to-face in-depth interviews were conducted using a purposive sampling method with patients enrolled in CCPs. In total, 64 participants aged between 41 to 94 years were recruited. A deductive framework was developed using the Picker Patient Experience instrument to guide our analysis. Inductive coding was also conducted which resulted in emergence of new themes.

**Results:**

Our findings highlighted eight key themes of patient experience: i) ensuring care continuity, ii) involvement of family, iii) access to emotional support, vi) ensuring physical comfort, v) coordination of services between providers, vi) providing patient education, vii) importance of respect for patients, and viii) healthcare financing.

**Conclusion:**

Our results demonstrated that patient experience is multi-faceted, and dimensions of patient experience vary according to healthcare settings. As most patient experience frameworks were developed based on a single care setting in western populations, our findings can inform the development of a culturally relevant instrument to measure patient experience of community-based care for a multi-ethnic Asian context.

## Introduction

Global population expansion and ageing have resulted in an increase in the number of people living with chronic conditions, and an overwhelming burden to healthcare infrastructures and budgets [1–3]. As a result, tertiary healthcare facilities in most countries are facing systemic stress from high bed occupancies, emergency department presentations, and readmission loads [4–6]. This unprecedented strain has created an urgency to provide care in the community for patients with stable chronic conditions, in order not to overwhelm resources at tertiary hospitals [7]. Transitional care programmes organise health and social care around patients for safe re-entry into the community for follow-up management [8]. As such, these programmes offer numerous advantages to a healthcare system, including the improvement of overall patient satisfaction, quality of life, clinical outcomes and cost containment, particularly through the attenuation of unplanned and avoidable hospital admissions [9–14]. Henceforth, there is increased emphasis on transitional care programmes which incorporate strategies that ensure optimal management of patients at all care interfaces [15, 16].

To ensure that transitional care programmes are optimally designed, experiences of patients enrolled in these programmes must be understood and enhanced. A meta-synthesis by Allen et al. (2016) had shown that positive patient experience served as levers for improving clinical outcomes and safety for multimorbid patients [17]. Positive patient experience is thus vital to the success of transitional care programmes as it augments the efficient usage of healthcare resources at all echelons of care [18, 19]. Furthermore, a systematic review by Doyle et al. (2013) found an association between positive patient experience and adherence to medical treatments, improved clinical effectiveness and outcomes in hospitals and primary care centres across various countries, including the United States of America (USA), United Kingdom (UK) and Taiwan [20]. Given the increased leverage and importance of such programmes, there is an impetus to determine the key dimensions of patient experience constituting a successful care transition.

### Context of the study

Like many developed countries, Singapore, a multi-ethnic urban state in Southeast Asia, is facing a greying population. The disproportionately high number of elderly is coupled with an inevitable rise in chronic disease burden and surge in demand for long-term care management [21]. About half of Singaporean residents aged 60 years and above had reported having multiple chronic conditions, leading to a steady increase in healthcare utilisation and cost [22, 23]. Therefore, healthcare systems in Singapore have had to rapidly adapt to provide care in the community for stable chronic patients so as to prevent potential strain on tertiary hospital resources. To achieve this, healthcare systems had to effectively facilitate the integration of patient care across multiple care facets and settings. Henceforth, the public healthcare institutions in the country were organised into three integrated healthcare clusters, each forming one of three Regional Health Systems (RHSs). Each RHS has a full suite of services spanning across different care settings and operated with the strategic vision of steering holistic patient-centred care for the population [24]. Our study utilised data from the National University Health System (NUHS), one of three integrated healthcare clusters in Singapore. NUHS is helmed by the National University Hospital (NUH), which works in close partnership with other regional providers such as primary care physicians, community hospitals and academic health sciences institutions in the Western region of Singapore [25].

With greater assimilation across providers, NUHS harnessed its network resources to provide community-based care, with the assurance that their community partners were well-equipped to manage these patients. To that end, NUHS implemented their community right-siting and post-discharge care programmes to permit seamless transition of chronic patients from hospital to community [26]. Community right-siting programmes refer to initiatives facilitating the timely discharge of clinically stable chronic patients who received care delivered by multiple specialists from Specialist Outpatient Clinics (SOCs) to suitable community partners [27]. Post-discharge care programmes refer to initiatives that manage patients with frequent admissions, in need of high acuity care, and/or are non-ambulatory to be managed by a care team after they have been discharged from hospital [28]. Hereinafter, transitional care programmes which encompass a board range of services and environment designed to promote safe and timely passage of patients across care venues [8], such as the community right-siting and post-discharge care programmes will be referred to as community-based care programmes (CCPs) (Details of CCPs are shown in S1 Table).

### Defining the patient experience

Understanding the dimensions of patient experience is paramount in capturing the patient experience accurately [29]. Despite the importance of patient experience, there is a dearth in consensus on the dimensions of patient experience for CCPs. Motives for the derivation of patient experience instruments and hence their dimensions across health systems and countries vary from capturing the accountability of healthcare providers to enhancing patient choice, improving quality of care or measuring the overall performance of a health system [30]. After a review of existing literature, we found that most patient experience instruments were validated in the western context and validated for use in a single care venue (Instruments reviewed in S2 Table). A critical inquiry performed by Wolf et al. (2014) was the only review we had found to have evaluated a wide range of sources delineating patient experience definitions which elucidated the overlapping dimensions across the sources as the overall well-being of the patient influenced by the interplay of personal, societal and institutional factors at all venues of care [31]. The review concluded that the definition derived by the Beryl Institute most accurately captured the core components of patient experience: the ‘*sum of all interactions*, *shaped by an organisations’ culture*, *that influences patients perceptions across the continuum of care’* [32].

### Measuring the patient experience

Using the dimensions explicated by Wolf et al. (2014) and the definition proposed by the Beryl Institute, we concluded that the Picker Patient Experience instrument (PPE-15), conceived by the Picker Institute Europe, was the most comprehensive in capturing the patient experience [33]. Furthermore, the PPE-15 has also been validated in Hong Kong, demonstrating its cultural relevance for deployment in an Asian city such as Singapore [34]. The PPE-15 dimensions include: i) information and education, ii) care coordination, iii) physical comfort, iv) emotional support, v) respect for patient preferences, vi) involvement of family and friends, and vii) continuity and transition [33]. To further confirm that there were no other instruments that better captured the dimensions than PPE-15, we created a comparison table to compare the instruments we had reviewed against the dimensions in PPE-15 (S3 Table).

### Rationale for understanding patient experience in the Asian context

At the patient level, expectations and values of Asian patients are vastly different from Western patients as ethnic and socio-cultural nuances influence the receptiveness to seek care and sticking to a management plan [35, 36]. Kai et al. (2011) also highlighted the cultural variations between Asian and European patients regarding patient autonomy and involvement of the family in information disclosure and decision making for cancer patients in the UK [37]. At the systems level, healthcare financing and provision arrangements are conceived as articulations of the broader institutional and philosophical contexts. The diversity of the healthcare topography in Asian countries might render the applicability of western concepts and propositions of patient experience sub-relevant [38].

To our knowledge, most studies were conducted in predominantly western populations and had examined the patient experience within the ambit of a particular care setting such as general practice, inpatient or outpatient clinics. Thus, there is a paucity of empirical studies exploring the patient experience of CCPs in the Asian context. Therefore, the aim of this study is to explore patients’ experiences with CCPs and develop dimensions of patient experience salient to CCPs in the Singapore context.

## Methodology

This qualitative study used data extracted from de-identified transcripts of interviews conducted as part of two other prior studies, whereby the evaluation of Post-Discharge Care Programmes (DSRB 2016–00410) and NUHS Right-Site Care Programmes (DSRB 2016–00914) were implemented by NUHS [39, 40]. A conceptual framework was subsequently used to analyse the transcripts for the derivation of themes salient patient experience for CCPs. We employed the Consolidated Criteria for Reporting of Qualitative Research (COREQ) throughout our study (S4 Table).

### Sampling and recruitment process

Face-to-face in-depth interviews were conducted using a purposive sampling method. Participants were eligible if they were able to (a) give informed consent or have proxies who could provide consent on their behalf; and (b) converse in English, Mandarin, Malay or Tamil. Exclusion criteria included participants with cognitive dysfunction or those without a representative to provide consent on their behalf; or those who refused to be audio recorded.

Semi-structured interview guides were utilised for this study (S1 Appendix). Data was collected between 8 July 2016 to 26 April 2018. The interviews took between 22 minutes to 98 minutes and were conducted at a place of the participants’ convenience. No repeat interview was carried out. The interviews were audio-recorded, transcribed verbatim and coded thematically. A total of 30 transcripts were translated to English, 24 from Mandarin, 5 from Malay and 1 from Tamil. All audio transcripts and collected field notes were de-identified to maintain participants’ anonymity. Member checking, whereby the interview transcripts were sent back to the participants to be reviewed, was not performed.

### Conceptual framework for analysis

We utilised the framework which was developed from PPE-15 to guide the initial coding process. Herein, we will summarise our data analysis procedure based on a five-step approach proposed by Ritchie and Spencer [41].

1. Familiarisation with data: Coders got acquainted with the data through reading and re-reading of transcripts. Notes were taken during the initial reading phase.
2. Identifying a framework: PPE-15 was concluded as most relevant for our analysis as it takes a comprehensive and holistic approach to the patient experience by covering all dimensions highlighted by Wolf et al. (2014).
3. Coding and indexing: We utilised the deductive framework which was developed from PPE-15 to guide the initial coding process. The key issues and concepts expressed by the participants formed additional themes and subthemes through an inductive coding process.
4. Charting: Specific pieces of data which were indexed in the previous stage were arranged into a framework matrix.
5. Mapping and interpretation: Associations and patterns within the data were elucidated for an accurate representation of the participants’ experience.

### Ethics board approval

Ethics approval was obtained from the National Healthcare Group, Singapore, Domain Specific Review Board (DSRB) before starting the study. The study reference number is 2018/00946.

## Results

### Summary of participants demographic characteristics

Table 1 summarizes the age, gender, and ethnicity of participants. In total, 64 participants aged between 41 to 94 years were recruited. Our participants have sufficiently represented the two genders and the three main ethnic groups in Singapore, namely Chinese, Malay and Indian, making our sample representative of a multi-ethnic population in Asia [42, 43].

**Table 1:** Characteristics of patients in community health programmes.

	N	Sample composition^1^ (%)
**Gender**		
Male	39	60.9
Female	25	39.1
**Age**		
41 to 50	6	9.4
51 to 60	8	12.5
61 to 70	12	18.8
71 to 80	23	35.9
81 to 90	9	14.1
91 to 100	6	9.4
**Ethnicity**		
Chinese	44	68.8
Malay	12	18.8
Indian	8	12.5

^1^ Total percentage might not sum to 100 due to rounding off to nearest one decimal place.

### Main findings

Our findings summarised in Table 2 revealed eight main themes and twelve additional subthemes that described the patient experience of patients enrolled in CCPs. When taken together, these themes fully encapsulate how the patients experienced their transitions across different care venues and the elements which they value. Not only were seven of the derived themes captured by PPE-15, a new theme, healthcare financing, was generated from the data during the inductive coding process.

**Table 2:** Main findings describing patient experiences of CCPs.

Themes	Subthemes
1. Ensuring care continuity	1.1 Familiarity with provider
	1.2 Empowered to act during emergencies
2. Involvement of family	2.1 Family’s involvement in decision-making
	2.2 Family’s involvement in caregiver duties
3. Access to emotional support	
4. Ensuring physical comfort	
5. Coordination of services between providers	5.1 Need for communication channels across care venues
	5.2 Need for consistent instructions to patients
6. Providing patient education	6.1 Explanation of disease and treatment options
	6.2 Provider’s perceptions of patient’s health literacy
7. Importance of respect for patients	7.1 Need for patient involvement in decision-making
	7.2 Lack of involvement in decision-making
8. Healthcare Financing	8.1 Eligibility for financial subsidies
	8.2 Need for clear explanation of financial subsidies by providers

### 1. Ensuring care continuity

The main theme of ensuring care continuity was reflected by participants as having care maintained throughout the patient journey. This was established by nurturing long-term patient-provider relationships when patients saw regular providers in the community post-discharge. A consistent relationship with the patients enabled providers to tailor individualised transitional care plans and instilled patients with the necessary skills for the self-management of their conditions. Ensuring care continuity was highlighted by two additional subthemes; 1.1 Familiarity with provider and 1.2 Empowerment to act in emergencies.

#### 1.1. Familiarity with provider

Patients who received care from the same provider in the community had a heightened sense of familiarity, ease and confidence in their follow-up care. For providers, augmented acquaintance with patients’ previous medical and personal histories enabled them to construct more long-term personalised care plans encompassing lifestyle recommendations and recovery strategies.

“*I feel that they are taking care of me, and with the same doctor, we also feel very confident*.”

(*IDI 51, Male, 65, Malay*)

“*[…] I can say it is better because I see a fixed doctor. Not like [hospital], whereby different doctors are on duty. So that particular doctor I see at [primary care clinic], he knows my condition well. He gives me almost 3–6 months visit [follow-up] […] there is a personal touch to that, not a bonding but a personal relationship with him […] advised me about my medical condition, that I should quit smoking, that I should lose weight. It is considered good in a way*.”

(*IDI 20, Male, 44, Chinese*)

#### 1.2. Empowered to act in emergencies

Providers equipped patients with the knowledge, confidence, and self-determination for their health as part of their management plan. This preparation enabled patients to appropriately address medical emergencies such as minor relapses or sudden deterioration in condition. Therefore, knowledge of symptom management and healthcare access to cope with simple complications when patients are in the community reduced unnecessary healthcare utilisation.

“*Previously, it [asthma] was more severe. Now it is milder, and I just use the inhaler at home. The nurses also said that there is no need to go to the hospital, just use the inhaler at home, and that is fine. I used the inhaler myself when it is severe, and it is fine*.”

(*IDI 6, Male, 73, Chinese*)

### 2. Involvement of family

The involvement of family members played a significant role in the decision-making process and was a key theme in this study. Participants emphasised how family members played a caregiving role for patients after the transition back home by acting as chaperones for patients to bridge the transition across care localities. Involvement of family was further highlighted with the following two subthemes; 2.2. Family’s involvement in decision-making and 2.2. Family’s involvement in caregiving duties.

#### 2.1. Family’s involvement in decision-making

Family members were devolved the responsibility to make decisions on care treatments, particularly for older patients who harboured the perception that their more educated children could better understand the technicalities of medical treatments. Patients’ children, therefore, served as conduits to translate the “medical intensive things” into simpler terms for patients to comprehend. Family members were also advised on the patient’s condition so that they could make more informed decisions on the patient’s management plan.

“*Actually, when I go [follow-up at primary care clinic] the past few times, my daughter was there. So certain things, technical, medical intensive things, [doctor] tell her, and she will explain it to me*.”

(*IDI 51, Male, 65, Malay*)

”*They [doctors] called for a meeting with all my family members since they had to test if it was benign or malignant then decide on the course of action. There were medications that could be taken to shrink the tumour, because if left it as it is, it might worsen […] They [doctors] called us [family] down to listen to what we [family] have to say and to let us know what they felt was best too*.”

(*IDI 38, Female, 92, Chinese*)

#### 2.2. Family’s involvement in caregiver duties

Family members were highly involved in certain aspects of post-hospital patient care. The duties included the monitoring of medication schedules, ensuring the consumption of correct medicine dosage which facilitated the completion of care processes post-discharge and responding to medical emergencies appropriately.

“*My daughter helps with the medication […] she will arrange the medication in terms of my weekly intake*.”

(*IDI 15, Male, 78, Chinese*)

“*Later at the night 12 o’clock, called my brother […] As a nurse, he will know what to do. Early in the morning 6 o’ clock, called my brother again, so my brother had to rush down [for the emergency] […] my brother also had to work, middle of the night 12 o’clock, morning 6 o’ clock, call him, he drives, it’s very distracting for him*.”

(*IDI 29, Male, 79, Chinese*)

### 3. Access to emotional support

Emotional care called for providers to confer compassionate care readily through discussing and allaying patients’ existing fears and anxieties. Participants revealed that the discharge process was also less intimidating when their healthcare providers and family members were present emotionally during the interactions and available for them after they re-enter the community.

“*He [nurse] also visited me at the ward to encourage me to get better soon [before transitioning into the community]*.”

(*IDI 7, Male, 74, Chinese*)

“*She [care coordinator] would often call us to see if we have any issues and that is sort of asking us about our well-being [matters] that we would like to talk to her about [patient is already in the community]*.”

(*IDI 60, Male, 65, Chinese*)

“*My younger brothers all have very nice wives. I have one brother’s wife, every day will call me [patient is already discharged]. Today, she got dialysis so she cannot call me […] My sisters, my brothers, they all love me so much*.”

(*IDI 56, Male, 65, Malay*)

### 4. Ensuring physical comfort

Prompt and calibrated pain management is a universal requirement for healthcare, necessitating providers to reduce patients’ pain during instances of discomfort and to complete medical procedures for the patient with the least amount of discomfort.

“*I tell my doctor I got some there [patient’s hand] got pain […] that day they found it and they jabbed the steroid*.”

(*IDI 23, Male, 59, Indian*)

“*The doctor also never tell me to and also never tell me to off the thing for one hour or give me painkiller. He just opened [undress the wound] like that. The first time it was opened, I shout like hell […] Sister [nurse’s name] came and said next time cannot open. Must wait for half an hour or one hour after giving you the painkiller. From that time onwards, I asked doctor do not open*.”

(*IDI 46, Male, 57, Malay*)

### 5. Coordination of services between providers

Care integration along the patient journey called for a degree of coordination between healthcare entities which entailed accurate dissemination of the patient’s management plan and instructions across care interfaces. Participants expressed that having clear channels for the sharing of information could have prevented the duplication of healthcare services and fragmentation of care. Therefore, coordination of services between providers was captured by the two subthemes; 5.1. Need for communication channels across care venues and 5.2. Need for consistent instructions to patients.

#### 5.1. Need for communication channels across care venues

Poor communication between providers from tertiary hospitals and community partners during referrals led to disjointed care management strategies. Community partner providers should have access to the full details regarding patient’s treatment plans to provide safe and effective post-hospital care.

“*The problem is that the doctor does not have full details of my records, my profile all during my stay in [care venue A] for the past few months. I had a few months stay at [care venue B], but that is only a few months […] do not have the full information of what [procedures] I had gone through at [care venue A]*.”

(*IDI 47, Male, 58, Malay*)

#### 5.2. Need for consistent instructions to patients

The provision of care management instructions from providers to patients shifting from tertiary hospitals to the community should be consistent to ensure that patients receive the same treatment and quality of care throughout their patient journey. Therefore, reciprocal communication between providers could have reduced conflicting instructions regarding medication regimens and other care processes, which made adherence to care plans confusing for patients.

“*Pharmacy say actually this one cannot take together with don’t know what medicine. Because before breakfast must take this thyroid medicine. Then do not know how many hours apart then can take that one, so another medicine cannot take together […] I found out myself, so when doctor mentioned something I said no, it is not like this*.”

(*IDI 41, Female, 94, Chinese*)

### 6. Providing patient education

The provision of timely and unambiguous information regarding patients’ disease conditions and management trajectories was discovered as an essential pre-requisite for patients to take the necessary steps to plan their care before transitioning into the community. The main theme, providing patient education was expanded by the two subthemes; 6.1. Explanation of disease and treatment options and 6.2. Provider’s perception of patient’s health literacy.

#### 6.1. Explanation of disease and treatment options

Providers furnished patients with information regarding their disease conditions such as the pathophysiology of disease, prospective medical procedures, or recommended medications. Presenting comprehensible and objective information aided patients in weighing the benefits and risks involved in selecting prospective treatment plans, which allowed patients to act on the information based on their best interest.

“*[Doctor] said that if there was a blockage of stools, it exerts pressure on the urinary tract and prevents from passing urine as well*.”

(*IDI 13, Male, 77, Chinese*)

“*If I take too much cholesterol medication, there is a chance I might contract cataract*.”

(*IDI 1, Male, 63, Chinese*)

“*They [doctors] told me first what the risks were. Then I said never mind I will do it*.”

(*IDI 55, Female, 72, Malay*)

#### 6.2. Provider’s perception of patient’s health literacy

Some providers harboured the impression that detailed explanations of the disease condition and treatment options were unnecessary due to their assumption that patients could not understand the medical jargon. Flawed perceptions of patients’ inability to comprehend the technicalities of their treatment plans eroded the shared decision-making authority.

“*[…] even though I asked a lot of them [doctors] about the medicine, how the vitamin D can be improved, they are telling about the sunlight; sunlight is one of the things, general way of talking*.”

(*IDI 51, Male, 65, Malay*)

### 7. Importance of respect for patients

Respect for patients’ preferences engendered their active engagement in the decision-making process for their plan of care. Furthermore, the consent process laid the foundation for the fiduciary relationship between patient and provider, enabling the patients to translate their values and priorities into decisions. This made importance of respect for patients a salient theme for patient experience in CCPs and it was encapsulated by the two subthemes; 7.1. Need for patient involvement in decision-making and 7.2. Lack of desire to be consulted on decision-making.

#### 7.1. Need for patient involvement in decision-making

Some providers did not respect patients’ autonomy in the decision-making process, whereby a certain aspect of the care plan was changed without prior consultation with the patient. Thus, providers in the community need to consult the patients before making any adjustments to the care plans especially when patients are on regimens carried forward from hospital settings. Patients have their own preferences and should be engaged prior to making any changes such that their goals and expectations are assimilated into the care plan.

“*I brought it over from here [hospital name]. I felt the medicine brought over from the hospital was changed by them so they will give me medicine from their side [community partner] […] So sometimes I feel that I have to get used to the medicine from this side [community partner]. But I wonder why they would like to change for me […] However, I have been taking medicine from this brand all the time. You ask me to make a switch suddenly, and I felt quite weird, but their pharmacy did not explain anything to us. They just make a change of medicine directly*.”

(*IDI 32, Female, 71, Chinese*)

#### 7.2. Lack of desire to be consulted on decision-making

Some patients took a passive role in decision-making as they viewed doctors as figures of authority to make treatment-related decisions on their behalf. Therefore, such instances of paternalistic medicine silenced the voices of patients and promoted unilateral decision-making by providers.

“*Even if you give me options, I also don’t know. So, you just tell me this medicine is for that, I just have to obey right? So there is not much choice there. It’s not like a buffet, where you can choose to eat this, you can choose to eat that*.”

(*IDI 20, Male, 44, Chinese*)

### 8. Healthcare financing

A patient’s financial circumstances and out-of-pocket medical expenditure might affect their perceived affordability of healthcare services which could determine their willingness to seek or continue treatment. This theme of healthcare financing was highlighted by most of our participants and was supported by the two subthemes; 8.1. Eligibility for financial subsidies and 8.2. Importance on educating patients on financial subsidies.

#### 8.1. Eligibility for financial subsidies

Financial assistance for healthcare expenses is subjected to means-testing criteria. Medical social workers assessed the level of government subsidies that a patient was eligible for when utilising selected healthcare services. Stringent means-testing guidelines ensured that the dispersed quantum is calibrated according to the patient’s financial situation. However, financial circumstances are relative, and means-testing thresholds did not accurately capture the true needs of the patient.

“*We applied, but we failed the means-testing. I think it is a bit unfair though I did not fight for it. I think that means-testing has some guidelines which disadvantaged me […] guidelines are drawn like this, and unfortunately, I fall through the cracks, hurt my feelings. I did apply, but I didn’t qualify because of means-testing*.”

(*IDI 14, Male, 77, Chinese*)

#### 8.2. Importance on educating patients on financial subsidies

A clear understanding of available subsidy packages was crucial in the care planning process from a financial standpoint so that patients would not miss out on the financial help available to them. Providers, in this case, medical social workers, should be equipped to advise patients regarding subsidy arrangements available to them.

“*Then later we asked them [social workers] about the discount [subsidy], but they didn’t explain it clearly […] they didn’t properly answer regarding the 50% discount […] even if we asked them, they were not able to answer very clearly*.”

(*IDI 32, Female, 71, Chinese*)

## Discussion

This study has provided preliminary evidence on the dimensions that can be ascribed for the patient experience of patients enrolled in CCPs in the Singapore context. Unlike other patient experience instruments validated for usage in a single care context, our study applied a validated framework (PPE-15) commonly used in hospital settings to study patient experience for CCPs. Our findings ascertained that many of the dimensions were transferrable to the patient experience for CCPs. These dimensions captured elements of care continuity for patients transitioning from hospital to community with adequate emotional and physical support. One way to achieve that was for ample service coordination between providers across the care spectrum. Patients also required a certain level of knowledge regarding their disease conditions and treatment options to effectively take part in the care planning process. Interestingly, we had discovered that there were differences in certain dimensions due to cultural distinctions between eastern and western developed frameworks, which makes our dimensions more relevant for deployment in the Asian context. The dimensions that had divergences included respect for patient’s preferences, involvement of family and healthcare financing. Hereinafter we will discuss the aforementioned dimensions with particular emphasis on elements salient to CCPs in the Asian context.

We concluded that the patient experience for community-based care programmes was indeed “a sum of all interactions” as explicated in the definition by the Beryl Institute [32]. In this case, reciprocal communication between providers across care venues and with patients, ensured that care was consolidated, and preferences of patients respected both at the hospital and community settings [44]. Perhaps more unique to the Asian context is the difference in patients’ autonomy when planning their transitional care as compared to their western counterparts. Asian patients tend to perceive doctors as figures of authority, consequently ceding power to make decisions to the provider [45]. Thus, the perceived power differentials between patient and doctor and hierarchal structure in Asian societies attributed to the low desire for shared decision making as seen in some of our participants [46, 47]. This contrasts with the desire for more autonomy as seen in Western patients [37].

Cultivating a certain level of patient activation is crucial before patients’ re-entry to the community to ensure that patients manage their emotional sequela and self-efficacy associated with their illness in the absence of a trained healthcare personnel [48, 49]. This can be achieved through provision of self-management knowledge, reinforcing the dimension that patient education should be integrated into transitional care planning [50]. Aside from empowering patients, caregivers such as family members should also be considered within the patient experience. As seen in our participants, family members were often the unofficially appointed caregiver and decision-maker. As a caregiver, family members need to be equipped with the relevant knowledge to execute the discharge instructions and react to medical emergencies especially during the absence of a healthcare provider when the patient has been discharged into the community [51–53]. Regarding decision-making, for Asian patients, family involvement is highly pertinent, especially for patients who hold strong to certain virtues such as Confucian doctrines, where filial piety asserts a strong influence on medical decision-making. Unlike the classical western concept of autonomy, in which decisions are expectantly made in the absence of external influences, the eastern concept of autonomy admits the need for ‘‘engagement and interactions in a network of relations with others” [54]. Therefore, decisions regarding the patient’s medical care are made at the familial level, given that such choices could have repercussions for family members [55]. The virtue of filial piety also places an obligatory duty on children to care for their elders throughout their patient journey, driven in part by an appreciation of the care they had previously received [56].

Financing is not a dimension that is reflected in most patient experience instruments. To our knowledge, only the Outpatient Experience Questionnaire (OPEQ) developed by China had captured the dimension of medical expenses [57]. This could be due to cultural factors and differences in configurations of healthcare systems. Both Singapore and China have a healthcare system that necessitates a certain level of out-of-pocket payment for health services utilisation [58–62]. From a cultural perspective, Chinese patients might exercise a more frugal mentality than their Western counterparts when it comes to any form of expenditure and healthcare is no exception [63]. This can be seen in our participants, where medical expenses are of huge concern. Despite various financial safety measures in place by the Singapore healthcare system, patients suffering from chronic diseases often face high and prolonged medical costs [64]. The inability to secure financial subsidies poses a threat to the patient experience as a patient’s financial situation affects their willingness to receive or continue treatment [65]. We concluded that the dimension of financing is particularly relevant to Asian countries whereby the culture of saving is firmly ingrained in the society due to philosophical underpinnings [66].

### Strengths and limitations

To our knowledge, this is the first qualitative study conducted which explicitly examines the patient experience for CCPs a multi-ethnic Asian context. We also had a large sample size of 64 participants with different sets of chronic conditions to ensure that the experiences of CCPs for patients with one condition does not differ drastically from another. Additionally, even though a conceptual framework was used to carry out the thematic analysis deductively, we coded for new themes salient to the patient experience so as to fully capture all elements of the patient experience. This allowed themes that were relevant to patient experience for CCPs but not present in the original PPE-15 framework to be elucidated.

Even though the number of participants recruited was representative of the ethnic distribution in Singapore, the sample was predominantly Chinese participants. Thus, our data might not fully capture the patient experiences of the Malay and Indian ethnic groups. A maximum proportion sampling of each ethnic group to explore any cultural differences in patient experience can be applied to future studies. In addition, participants might have recall bias as they were interviewed at different time points during their transitional care journeys. We also acknowledge that there might be a potential variation in the patient experience for patients of different age groups due to differences in various needs such as perceptions and expectations of healthcare interactions and experiences [67, 68]. Lastly, our sample consisted of more males (60.9%) as compared to females (39.1%), as more males in Singapore suffer from chronic conditions [69]. Differences in patient experience between genders and age groups can be explored in future studies.

### Implications

As most developed countries shift their healthcare services from hospital to community, a safe and seamless transition is central for patients who require chronic care to re-enter the community with a certain level of patient activation. With unique healthcare structures and diasporas distinctly different from the West, the development of a patient experience instrument for the Asian context with the dimensions derived from this study is justified. Based on our study findings, we propose future research to prioritise deriving an operational definition for patient experience of patients enrolled in CCPs and developing a culturally relevant instrument to measure patient experience for such programmes to create a common measure and understanding of patient experience. To achieve this, follow-up works using a Delphi method can be employed, where multiple rounds of controlled opinion feedback can be sought from experts to reach a consensus on the definition of patient experience and to validate the appropriateness of items in a patient experience instrument which employs dimensions earmarked from this study [70].

## Conclusion

This study explored the dimensions of patient experience associated with CCPs and elucidated the differences in certain areas of patient experience accruing to the cultural nuances between care contexts. As a result, we ascertained that even though dimensions are largely consistent across care contexts, the design and implementation of CCPs should take into account cultural variations to ensure a positive patient experience.

## Supporting information

S1 Table Summary of community-based care programmes by NUHS. (DOCX)

S2 Table Existing patient experience instruments. (DOCX)

S3 Table Comparison of dimensions from patient experience instruments against PPE-15. (DOCX)

S4 Table COREQ checklist. (DOCX)

S1 Appendix Topic guides. (DOCX)
