# Supplementary material for: Exploring the dimensions of patient experience for community-based care programmes in a multi-ethnic Asian context
Source: PLoS One. 2020 Nov 25;15(11):e0242610. doi: 10.1371/journal.pone.0242610 (PMC7688169; doi:10.1371/journal.pone.0242610)
Supplement: S1 Table — (DOCX) [file pone.0242610.s001.docx]

**S1 Table. Summary of Community-based Care Programmes by NUHS.**

|  | **Programme** | **Description** |
| --- | --- | --- |
| Management of Frequent Admitters through Case Management | Aged Care Transition (ACTION) Team | Provision of home visits and monitoring of frequent admitters, who will be referred to befrienders and volunteers when needed. |
|  | The Airway Programme Team | Management of patients with chronic obstructive pulmonary disease. |
|  | National University Cancer Institute (NCIS) Team | Management of cancer patients by oncology nurses and specialised home care services. |
|  | St Luke’s Hospital (SLH) Team | Management of patients, who are discharged from ACTION Team or SLH, at St Luke’s Outpatient Clinic by a multidisciplinary team (including care coordinators, medical social workers and doctors). |
| Timely Discharge from Acute Hospital through Transition Care | NUH (National University Hospital) 2Home Programme | Provision of individualised needs assessment for patients before their discharge. |
|  | Heart Failure Programme | Provision of cardiac-specific services for patients with heart failure. |
|  | Negative Pressure Wound Therapy Programme | Provision of vacuum dressing, home visits, financial counselling and referral to medical social workers (when needed) for patients with clinical need. |
|  | Early Support Discharge Programme | Provision of home rehabilitation sessions for stable stroke patients. |
|  | Caring across Cancer Care Continuum Programme | Provision of care for cancer patients with National University Cancer Institute, Singapore (NCIS) Team. |
| Timely Discharge from Specialist Outpatient Clinic | - | Management of patients with less complicated chronic conditions by a primary care physician. |
| Delivery of Patient-Centered and Seamless Care | - | Management of patients with more complex chronic conditions by a consolidated specialist care clinic. |
