# Supplementary material for: Exploring the dimensions of patient experience for community-based care programmes in a multi-ethnic Asian context
Source: PLoS One. 2020 Nov 25;15(11):e0242610. doi: 10.1371/journal.pone.0242610 (PMC7688169; doi:10.1371/journal.pone.0242610)
Supplement: S2 Table — (DOCX) [file pone.0242610.s002.docx]

**S2 Table. Existing patient experience instruments.**

| **Name of instrument** | **Brief description of the questionnaire** | **Core dimensions of the questionnaire** |
| --- | --- | --- |
| Care Transition Measure (CTM) (71,72) | A 3-item and 15-item tool that measures the perceived quality of care transition to ensure the quality and coordination of care as patients transition between different locations and levels of care. | 1. Information transfer 2. Patient and caregiver preparation 3. Self-management support 4. Empowerment to exert preferencs |
| Partners at Care Transitions Measure (PACT-M) (73) | A piloted tested patient reported questionnaire for the evaluation of the quality and safety of care transitions from hospital to home for older individuals in the UK. | 1. Patient involvement 2. Medication management 3. Discharge arrangements 4. Coordination with other providers 5. Providing information and guidance to patient/ family 6. Providing psychological and social support 7. Anticipation and preparation for emergencies/ deterioration 8. Feeling of safety |
| HowRwe Questionnaire (74) | A short generic questionnaire consisting of four questions that is suitable for frequent use across all health and social care sectors. It aimed to reduce respondent burden, enable rapid feedback and comparisons to be made between different care settings either within an organisation or across the patient pathway. | 1. The kindness of the provider towards patient 2. Communication by the provider to patient 3. Promptness in providing service to the patient 4. Organisation of services |
| Generic Short Patient Experiences Questionnaire (GS-PEQ) (75) | The Generic Short Patient Experiences Questionnaire (GS-PEQ) is a short, generic set of ten questions on user experiences with specialist healthcare that covers important topics for a range of groups. | 1. Communication with providers 2. Patient’s confidence in the provider 3. Information provided to the patient 4. Personalised care plan 5. Patient’s involvement in decision-making 6. Organisation of care 7. Waiting time 8. Overall satisfaction 9. Perceived benefits 10. Perceived mistakes made by providers |
| Consumer Quality Index (CQI) Inpatient Hospital Care (76) | A 50-item questionnaire validated in the Dutch context to evaluate inpatient experience at both the hospital and individual department level. | 1. Admission 2. Communication with nurses 3. Communication with doctors 4. Own contribution 5. Explanation of treatment 6. Pain management 7. Communication about medication 8. Feeling of safety 9. Information at discharge |
| Warwick Patient Experiences Framework (WaPEF) (77) | The WaPEF was the first patient experiences framework with an explicit link to an underpinning patient evidence base, linking themes and sub-themes with specific references. The WaPEF informed the structure and content of the NICE Patient Experiences Guidance. | 1. Patient as an active participant 2. Responsiveness of services 3. Lived experience 4. Continuity of care and relationships 5. Communication 6. Information 7. Support |
| Hospital Consumer Assessment of Healthcare Providers and Systems (HCAHPS) (78) | A 32-item instrument was designed to 1) produce comparable data on the patient’s perspective on care that allows objective and meaningful comparisons between hospitals on domains that are important to consumers and 2) create incentives for hospitals to improve their quality of care. | 1. Communication with providers 2. Responsiveness of providers 3. Pain management 4. Communication about medication 5. Hospital facilities 6. Discharge information 7. Overall rating |
| Patient Assessment of Chronic Illness Care (O-PACIC) Scale (79) | A 20-item instrument that to access the implementation of the Chronic Care Model from the patient’s perspective that focuses on patient-centred care and self-management behaviours. | 1. Patient activation 2. Delivery system/ practice-design 3. Goal setting/ tailoring 4. Problem-solving/ contextual 5. Follow-up/ coordination |
| Patient Experience of Integrated Care Scale (PEICS) (80) | A 17-item scale that measures patient experience of integrated care in a population with chronic conditions seen in primary care. | 1. Consideration of patient and family needs 2. Communication with the patient and between practitioners 3. Access to information 4. Involvement in decision-making 5. Care planning 6. Transitions between various health professionals and practitioners |
| Nordic Patient Experience Questionnaire (NORPEQ) (81) | An 8-item questionnaire that was developed in Norway to be a brief measure of the inpatient encounter. | 1. Doctors understandable 2. Doctors’ professional skills 3. Nurses’ professional skills 4. Nursing care 5. Health personnel interested in the problem 6. Information on tests |
| Norwegian Patient Experiences with GP Questionnaire (PEQ-GP) (82) | A 30-item questionnaire developed in Norway for the assessment of patient experiences for healthcare encounters with general practitioners. | 1. General practitioner’s medical and relational skills 2. Organisation of the practice and assessment of auxiliary staff 3. Acceptability of waiting time |
| Outpatient Experience Questionnaire (OPEQ) (83) | The OPEQ was developed as a tool to measure the outpatient experiences in comprehensive public hospitals in China. | 1. Physical environment and convenience 2. Doctor-patient communication 3. Health information 4. Medical expenses 5. Short-time outcome 6. General satisfaction |
| Hong Kong Inpatient Experience Questionnaire (HKIEQ) (84) | The HKIEQ was designed in Hong Kong based on the Picker Patient Experience-15 Questionnaire developed in the United Kingdom. | 1. Access, choice, coordination 2. Communication and information 3. Privacy 4. Involvement in decisions 5. Physical comfort and pain relief 6. Environment and facilities 7. Involvement of family and friends 8. Support for self-care 9. Care of healthcare professionals and feedback handling |
| Patient Experiences Questionnaire (PEQ) (85) | The PEQ is a self-reported instrument containing 35 items developed to cover the most important aspects that patients value in the inpatient hospital setting. | 1. Information future complaints 2. Nursing services 3. Communication 4. Contact with next-of-kin 5. Doctor services 6. Hospital and equipment 7. Information medication 8. Organisation 9. General satisfaction |
| Picker Patient Experience-15 (PPE-15) (33) | The PPE-15 was developed to elicit feedback from patients to highlight aspects of care that needed improvement and to monitor the quality of care. It consists of 15 questions which measure eight dimensions of care. | 1. Information and education 2. Physical comfort 3. Involvement of family and friends 4. Emotional support 5. Continuity and transition 6. Coordination of care 7. Respect for patient’s preferences 8. Overall impression |

**References**

71. Coleman EA, Smith JD, Frank JC, Eilertsen TB, Thiare JN, Kramer AM. Development and testing of a measure designed to assess the quality of care transitions. Int J Integr Care. 2002;2:e02.

72. Parry C, Mahoney E, Chalmers SA, Coleman EA. Assessing the Quality of Transitional Care: Further Applications of the Care Transitions Measure. Med Care. 2008;46(3):317–22.

73. Oikonomou E, Chatburn E, Higham H, Murray J, Lawton R, Vincent C. Developing a measure to assess the quality of care transitions for older people. BMC Health Serv Res. 2019 Dec;19(1):1–12.

74. Benson T, Potts HW. A short generic patient experience questionnaire: howRwedevelopment and validation. BMC Health Serv Res. 2014 Oct 22;14(1):499.

75. Sjetne IS, Bjertnaes OA, Olsen RV, Iversen HH, Bukholm G. The Generic Short Patient Experiences Questionnaire (GS-PEQ): identification of core items from a survey in Norway. BMC Health Serv Res. 2011 Apr 21;11(1):88.

76. Smirnova A, Lombarts KMJMH, Arah OA, van der Vleuten CPM. Closing the patient experience chasm: A two-level validation of the Consumer Quality Index Inpatient Hospital Care. Health Expect Int J Public Particip Health Care Health Policy. 2017;20(5):1041–8.

77. Staniszewska S, Boardman F, Gunn L, Roberts J, Clay D, Seers K, et al. The Warwick Patient Experiences Framework: patient-based evidence in clinical guidelines. Int J Qual Health Care. 2014 Apr 1;26(2):151–7.

78. Giordano LA, Elliott MN, Goldstein E, Lehrman WG, Spencer PA. Development, Implementation, and Public Reporting of the HCAHPS Survey. Med Care Res Rev. 2010 Feb 1;67(1):27–37.

79. Schmittdiel J, Mosen DM, Glasgow RE, Hibbard J, Remmers C, Bellows J. Patient Assessment of Chronic Illness Care (PACIC) and Improved Patient-centered Outcomes for Chronic Conditions. J Gen Intern Med. 2008 Jan;23(1):77–80.

80. Joober H, Chouinard M-C, King J, Lambert M, Hudon É, Hudon C. The Patient Experience of Integrated Care Scale: A Validation Study among Patients with Chronic Conditions Seen in Primary Care. Int J Integr Care. 2018 Oct 12;18(4):1.

81. Oltedal S, Garratt A, Bjertnæs Ø, Bjørnsdottìr M, Freil M, Sachs M. The NORPEQ patient experiences questionnaire: Data quality, internal consistency and validity following a Norwegian inpatient survey. Scand J Public Health. 2007 Oct 1;35(5):540–7.

82. Holmboe O, Iversen HH, Danielsen K, Bjertnaes O. The Norwegian patient experiences with GP questionnaire (PEQ-GP): reliability and construct validity following a national survey. BMJ Open. 2017 Sep 1;7(9):e016644.

83. Garratt AM, Bjaertnes ØA, Krogstad U, Gulbrandsen P. The OutPatient Experiences Questionnaire (OPEQ): data quality, reliability, and validity in patients attending 52 Norwegian hospitals. Qual Saf Health Care. 2005 Dec;14(6):433–7.

84. Wong ELY, Coulter A, Cheung AWL, Yam CHK, Yeoh EK, Griffiths S. Validation of inpatient experience questionnaire. Int J Qual Health Care. 2013 Sep 1;25(4):443–51.

85. Pettersen KI, Veenstra M, Guldvog B, Kolstad A. The Patient Experiences Questionnaire: development, validity and reliability. Int J Qual Health Care. 2004 Dec 1;16(6):453–63.
