# Supplementary material for: Exploring the dimensions of patient experience for community-based care programmes in a multi-ethnic Asian context
Source: PLoS One. 2020 Nov 25;15(11):e0242610. doi: 10.1371/journal.pone.0242610 (PMC7688169; doi:10.1371/journal.pone.0242610)
Supplement: S3 Table — (DOCX) [file pone.0242610.s003.docx]

**S3 Table. Comparison of dimensions from patient experience instruments against PPE-15**

| **Patient Experience Instruments** | **PPE-15 Dimensions** | | | | | | |
| --- | --- | --- | --- | --- | --- | --- | --- |
|  | Continuity and transition | Involvement of family and friends | Emotional support | Physical comfort | Care coordination | Information and education | Respect for patient preferences |
| Care Transition Measure (CTM) | **✓** | **✓** |  |  |  | **✓** | **✓** |
| Partners at Care Transitions Measure (PACT-M) | **✓** | **✓** | **✓** |  | **✓** | **✓** | **✓** |
| HowRwe Questionnaire |  |  | **✓** |  |  | **✓** | **✓** |
| Generic Short Patient Experiences Questionnaire (GS-PEQ) |  |  |  |  | **✓** | **✓** | **✓** |
| Consumer Quality Index (COI) Inpatient Hospital Care | **✓** |  |  | **✓** | **✓** | **✓** | **✓** |
| Warwick Patient Experiences Framework (WaPEF) | **✓** |  | **✓** | **✓** |  | **✓** | **✓** |
| Hospital Consumer Assessment of Healthcare Providers and Systems (HCAHPS) | **✓** |  |  | **✓** |  | **✓** | **✓** |
| Patient Assessment of Chronic Illness Care (PACIC) | **✓** |  |  |  |  | **✓** | **✓** |
| Patient Experience of Integrated Care Scale (PEICS) | **✓** | **✓** |  |  | **✓** | **✓** | **✓** |
| Nordic Patient Experience Questionnaire (NORPEQ) |  |  |  |  |  | **✓** |  |
| Norwegian Patient Experiences with GP Questionnaire (PEQ-GP) |  |  |  |  | **✓** | **✓** | **✓** |
| Outpatient Experience Questionnaire (OPEQ) | **✓** |  | **✓** |  |  | **✓** | **✓** |
| Hong Kong Inpatient Experience Questionnaire (HKIEQ) | **✓** | **✓** | **✓** | **✓** | **✓** | **✓** | **✓** |
| Patient Experiences Questionnaire (PEQ) | **✓** | **✓** |  | **✓** |  | **✓** | **✓** |
