# Supplementary material for: Exploring the dimensions of patient experience for community-based care programmes in a multi-ethnic Asian context
Source: PLoS One. 2020 Nov 25;15(11):e0242610. doi: 10.1371/journal.pone.0242610 (PMC7688169; doi:10.1371/journal.pone.0242610)
Supplement: S4 Table — (DOCX) [file pone.0242610.s004.docx]

**S4 Table. COREQ Checklist**

| **No. Item** | **Details** |
| --- | --- |
| **DOMAIN 1: RESEARCH TEAM AND REFLEXIVITY** | |
| ***Personal characteristics*** | |
| 1. Interviewer/facilitator | N.A. (Secondary data from prior studies) |
| 2. Credentials (of coder, CD, YL) | Master of Public Health Graduate |
| 3. Occupation (of coder, CD, YL) | Research Associate |
| 4. Gender (of coder, CD, YL) | CD: Male, YL: Female |
| 5. Experience and training (of coder, CD, YL ) | CD: 2 years of prior experience in qualitative research, YL: 1 year of prior experience in qualitative research |
| ***Relationship with participants*** | |
| 6. Relationship established | No prior relationship with interviewers |
| 7. Participant knowledge of the interviewer | No prior knowledge with interviewers |
| 8. Interviewer characteristics | N.A. (Secondary data from prior studies) |
| **DOMAIN 2: STUDY DESIGN** | |
| ***Theoretical framework*** | |
| 9. Methodological orientation and theory | Qualitative exploratory method employing a conceptual framework analysis approach |
| ***Participant selection*** | |
| 10. Sampling | Purposive sampling |
| 11. Method of approach | Recruitment through a case manager |
| 12. Sample size | 64 participants |
| 13. Non-participation | 21 non-participants |
| ***Setting*** | |
| 14. Setting of data collection | Place of participants convenience |
| 15. Presence of non-participants | None |
| 16. Description of sample | Sample is sufficiently large and representative of Singapore’s ethnic distribution |
| ***Data collection*** | |
| 17. Interview guide | S1 Appendix |
| 18. Repeat interviews | Not conducted |
| 19. Audio/visual recording | Audio recording performed |
| 20. Field notes | Field notes were collected during the interviews |
| 21. Duration | 22 to 98 minutes for the interviews |
| 22. Data saturation | Data saturation was reached when no new themes emerged after coding 64 transcripts |
| 23. Transcripts returned | Transcripts were not returned |
| **DOMAIN 3: ANALYSIS AND FINDINGS** | |
| ***Data analysis*** | |
| 24. Number of data coders | Two coders (CD, YL) |
| 25. Description of the coding tree | Summarised in Table 2 |
| 26. Derivation of themes | Summarised under conceptual framework for analysis |
| 27. Software | QSR NVIVO version 12 |
| 28. Participant checking | Participant checking was not performed |
| ***Reporting*** | |
| 29. Quotations presented | Results section |
| 30. Data and ﬁndings consistent | Results and discussion sections |
| 31. Clarity of major themes | Results and discussion sections |
| 32. Clarity of minor themes | Results section |
