# Supplementary material for: Exploring the dimensions of patient experience for community-based care programmes in a multi-ethnic Asian context
Source: PLoS One. 2020 Nov 25;15(11):e0242610. doi: 10.1371/journal.pone.0242610 (PMC7688169; doi:10.1371/journal.pone.0242610)
Supplement: S1 Appendix — (DOCX) [file pone.0242610.s005.docx]

**S1 Appendix. Topic Guides.**

**Topic Guide A**

**Aim:** To examine patients’ or caregivers’ experiences with NUHS Post Discharge Care Programs.

**Respondents:** Patients or proxies who are enrolled into the NUHS Post Discharge Care Programs.

[Note to interviewer]: tell participant that the questions will be asked in relation to services provided by the NUHS Post Discharge Care Programs – mentioned the names of the case managers if you know

1. **Care Continuity**

- When you were discharged from the hospital, how were you or [patient’s name] *prepared for your transition*?
  - Did your healthcare providers explain to you about your conditions?
    - If yes, what did they explain to you?
  - Did your healthcare providers explain how your conditions should be managed?
    - If yes, what did they tell you about ways to manage your conditions?
  - Were you or [patient’s name] informed of the next step(s) of your care?
    - If yes, how have you been kept informed?
    - Is there a care plan?
- How were you *followed up*?
  - Who did you or [patient’s name] contact when you have any problem/question after your discharge from the hospital?
  - How did you or [patient’s name] communicate with your providers?
  - How have your providers been responding to you or [patient’s name]?
  - Did you get help when you needed?
    - What makes you say so?

1. **Care Coordination**

- Is there a person who helps you or [patient’s name] organize your healthcare?
  - If yes, would you please tell me more about this person?
- How was your *care arranged* for you or [patient’s name]?
  - Did your care manager refer you or [patient’s name] to the relevant community service providers such as GPs, befriender services etc?
    - If yes, please tell me more about it.
  - Did your case manager arrange your subsequent appointments?
    - If yes, please tell me more about it.
  - Did your case manager help you or [patient’s name] organize your medications?
    - If yes, please tell me more about it.

1. **Person-Focused Care**
   - Would you or [patient’s name] like to be *involved* in your care?
     - - How would you like to be involved in your healthcare?
   - How have you and family members been *involved i*n your treatment?
     - - Have you or [patient’s name] been actively engaging your providers in discussions about your condition?
       - Were you or [patient’s name] *involved in the* selection of your treatment *options*? Were you given choices?
       - How were you or [patient’s name] and *family members* involved in making decisions during your care?
       - How were you or [patient’s name] involved in the management of your conditions?
   - What kinds of help have you and your family members received from your case manager [CM’s name]?
     - Have you received sufficient support from them? What makes you say that?
     - Did your CM ask how your condition affect your life (physically and emotionally)?
       - In the occasions when you or [patient’s name] have some emotional problems, how did your CM [CM’s name] help you cope?
       - What did your case manager do to help you or [patient’s name] overcome difficulties you faced?
2. **Empowerment:**

- What *information* was provided to you by your case manager [CM’s name]?
  - Did he/she provide you or [patient’s name] with information about your conditions? Please tell me more about it.
  - Did he/she inform you or [patient’s name] about the ways to manage your condition at home? Please tell me more about it.
  - Did he/she provide you or [patient’s name] with information about how to identify symptoms to look out for? Please tell me more about it.
  - Did he/she provide you or [patient’s name] with information about the medications you were to take at home? Please tell me more about it.
  - Did he/she provide you or [patient’s name] with information about the medications side effects? Please tell me more about it.
  - Did he/she inform you or [patient’s name] about the contacts that you can reach out to? Please tell me more about it.
  - Did he/she inform you or [patient’s name] about the other services that are available for you at the community? Please tell me more about it.
- How has the information provided to you affected your care?
  - Was the information provided to you or [patient’s name] appropriate?
    - What makes you say that?
  - Was it useful to help you or [patient’s name] manage your conditions?
    - What makes you say that?
  - Was it useful to help you or [patient’s name] make informed decision and choices about your care and support?
    - What makes you say that?

1. **Care integration**
   - In general, do your healthcare providers including doctors, nurses, therapists, social workers and case managers work together? Please tell me how they work together
2. **Satisfaction**

- On a scale from 1-10 how satisfied are you or [patient’s name] with the care received?
  - What makes you give this rating?
- Would you or [patient’s name] recommend the care received to your friends or family or use the same care as received if you are offered it in the future?
- What do you or [patient’s name] like best about the service provided by the [name of CM or program]?
- What do you or [patient’s name] not like about the service provided by the [name of CM or program]?
- How can it be improved?

1. **Any other comments?**

**Topic Guide B**

**Aim:** To examine patients’ [proxy] experience of the NUHS right-site care programmes.

**Participants:** Patients or proxies who are enrolled into the NUHS right-site care programmes

Note to Interviewer:

- To complete screening questionnaire and demographic questionnaire at the start of the interview.
- Tell participant that the questions will be asked in relation to services provided by the NUHS right-site care programmes.

1. **Care Coordination**
   - How was your *care arranged* for you or [patient’s name]?
     - How were your appointments arranged?
     - How were your medications organised?
     - In the event you require other services, how did you obtain the services?
       - Please tell me more about it.
   - Is there someone who helps you or [patient’s name] organize your healthcare?
     - If yes, would you please tell me more about this person?
2. **Care Continuity**
   - How is your relationship with your healthcare providers (within NUH as well as in the community?
     - Do you see the same healthcare providers?
   - How were you or [patient’s name] *prepared for your transition to the community care provider*?
     - What information was provided to you?
       - Did your healthcare providers explain to you about your conditions?
       - Did your healthcare providers explain how your conditions should be managed?
       - Were you or [patient’s name] informed of the next step(s) of your care?
         - If yes, how have you been kept informed?
         - Is there a care plan?
       - Did he/she provide you or [patient’s name] with information about how to identify symptoms to look out for? Please tell me more about it.
       - Did he/she provide you or [patient’s name] with information about the medications you were to take at home? Please tell me more about it.
         - Did he/she provide you or [patient’s name] with information about the medications side effects? Please tell me more about it.
       - Were you or [patient’s name] informed of ways to contact your healthcare providers when you have any problem/question?
         - Please tell me about it.
       - Did he/she inform you or [patient’s name] about the other services that are available for you at the community? Please tell me more about it.
   - How were you *followed up*?
     - How did you or [patient’s name] communicate with your providers?
     - Who did you or [patient’s name] contact when you have any problem or question?
     - Did your primary care provider understand your conditions?
       - What make you say that?
     - How have your providers been responding to you or [patient’s name] problems or questions?
     - Did you get help when you needed?
       - What makes you say so?
3. **Person-Focused Care**
   - Would you or [patient’s name] like to be *involved* in your care?
   - How have you and family members been *involved i*n your treatment?
     - Have you or [patient’s name] been actively engaging your providers in discussions about your condition?
     - Were you or [patient’s name] *involved in the* selection of your treatment *options*? Were you given choices?
     - How were you or [patient’s name] and *family members* involved in making decisions during your care?
     - How were you or [patient’s name] involved in the management of your conditions?
     - How else you like to be involved in your healthcare?
   - What kinds of help have you and your family members received from your healthcare providers to manage your conditions?
     - Have you received sufficient support from them? What makes you say that?
     - Did your healthcare providers ask how your condition affects your life (physically and emotionally)?
     - In the occasions when you or [patient’s name] have some emotional problems, how did your healthcare providers help you cope?

o What did your healthcare providers do to help you or [patient’s name] overcome difficulties you faced?

- - - Were *your needs (medical, psychological and social) adequately* throughout your treatment period?

o What makes you say that?

1. **Empowerment:**
   - How has the service provided to you affected your care?
     - Was the information provided to you or [patient’s name] appropriate?
       - What makes you say that?
     - Was it useful to help you or [patient’s name] manage your conditions?
       - What makes you say that?
     - Was it useful to help you or [patient’s name] make informed decision and choices about your care and support?
       - What makes you say that?
2. **Care integration**

- In general, do your healthcare providers including doctors, nurses, therapists, social workers and care coordinators work together? Please tell me how they work together

1. **Satisfaction**

- On a scale from 1-10 how satisfied are you or [patient’s name] with the care received (explain more about the whole process)?

o What makes you give this rating?

- Would you or [patient’s name] recommend the care received to your friends or family or use the same care as received if you are offered it in the future?
- What do you or [patient’s name] like best about the right-site care programmes?
- What do you or [patient’s name] not like about the right-site care programmes?
- How can it be improved?

1. **Any other comments?**
